# Enhanced Electromechanical Performance of Dielectric Elastomer by Co-Crosslinking of Silane-Functionalized TiO_2_ with Polyacrylate

**DOI:** 10.3390/polym18070872

**Published:** 2026-04-01

**Authors:** Lingxiao Peng, Wenjie Si, Yuhui He, Nanying Ning, Jianfeng Wang

**Affiliations:** 1 State Key Laboratory of Organic-Inorganic Composites, Beijing University of Chemical Technology, Beijing 100029, China; 15343391802@163.com (L.P.); sw226284@gmail.com (W.S.); 2 Department of Urology, China-Japan Friendship Hospital, Beijing 100029, China; byhyh2016@163.com

**Keywords:** dielectric elastomer (DE), polyacrylate rubber, TiO_2_, co-crosslinking, electromechanical performance

## Abstract

Dielectric elastomer actuators (DEAs) are attracting much attention as candidates for next-generation flexible actuation. Among various DE matrices, polyacrylate rubber (AR) is especially promising owing to their intrinsically high dielectric constant (*ε*_r_) and good mechanical performance. In particular, its mechanical behavior is close to that of porcine bladder tissue, making it a potentially good material for soft biomedical actuators for artificial bladder constructs. To achieve high actuated strain, which requires high *ε*_r_, high breakdown strength, and low elastic modulus, an AR DE composite filled with silane-functionalized TiO_2_ was fabricated, exhibiting good electromechanical performance enabled by strengthened interfacial polarization. To improve compatibility between TiO_2_ and AR matrix, TiO_2_ was preferentially modified with a silane coupling agent (CA) that features a double bond as its functional group, which can be introduced on TiO_2_ surface and participate in vulcanization with AR, thereby forming co-crosslinking bridges that strengthen interfacial bonding, improve filler dispersion, and increase interfacial polarizability within the matrix. As a result, at relatively low filler loadings, the composite exhibits a significantly increased *ε*_r_, while maintaining low modulus, low dielectric loss and high elasticity. The 10 CA@TiO_2_/AR composite exhibits a maximal actuated strain of 7.9% at 31.9 kV/mm without pre-stretch, which is 1.48 times that of pure AR and 1.32 times that of the 10 TiO_2_/AR composite.

## 1. Introduction

**Figure 1:**
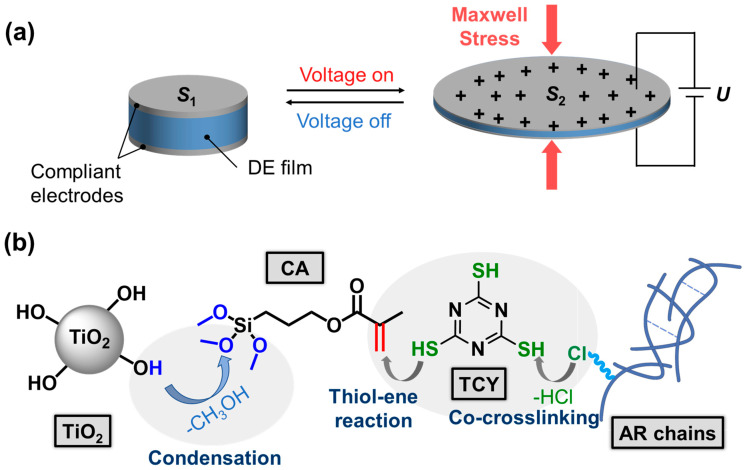
(**a**) The construction and electrical actuation mechanism of a typical DEA. (**b**) Schematic illustration of the fabrication method for CA@TiO_2_/AR composites.

Flexible actuators capable of generating large, rapid, and energy-efficient deformation are pivotal for the next-generation of microrobots, wearable and biomedical devices, and adaptive optical systems [1,2]. Among electroactive polymer technologies, dielectric elastomer actuators (DEAs) have emerged as a leading option thanks to their lightweight, large actuated strain, high output force, and remarkable energy density [3,4]. These characteristics make them suitable for use in artificial bladder applications, offering potential therapeutic benefits for patients with bladder dysfunction by helping restore normal urinary storage and release capabilities. A DEA comprises a flexible dielectric elastomer (DE) film sandwiched between compliant electrodes (Figure 1a). When a high voltage is applied, the resulting Maxwell stress compresses the film in thickness and drives in-plane expansion, directly converting electrical input into mechanical work. The actuated strain (*S*_a_) of a DE can be evaluated using the widely accepted theory proposed by Perline et al. [5].

(1) $$S_{a}=-\frac{P}{Y}=-\frac{\varepsilon_{0}\varepsilon_{r}E^{2}}{Y}$$

where *ε*_0_ is the vacuum permittivity, *ε*_r_, and *Y* are the dielectric constant and elastic modulus of DE, respectively, and *E* is the applied electric field. According to Equation (1), an ideal DE with excellent electromechanical performance should possess a high *ε*_r_ and a low *Y*, which together yield high electromechanical sensitivity (*β* = *ε*_r_/*Y*), as well as a high electrical breakdown strength (*E*_b_) [6,7]. Such an ideal DE can further be adapted for artificial bladder applications, where large, rapid, and reliable long-term actuation performance is essential.

Thanks to increased chain mobility from plasticizers, the *Y* of DEs can be reduced by introducing additives such as polyethylene glycol [8], silicone oil [9], epoxidized soybean oil [10], and ionic liquid [11] into the polymer matrix. For example, Ruan et al. used tri-*n*-butyl phosphate to plasticize brominated butyl rubber (BIIR), which weakened intermolecular interactions among BIIR chains and disrupted the filler network, thereby lowering *Y* [12]. However, introducing plasticizers can cause viscoelastic issues such as creep, stress relaxation and increase hysteresis loss [13]. In addition, limited compatibility with many polymer matrices promotes migration of plasticizer molecules and shortens device service life.

Beyond mechanical softening strategies, increasing *ε*_r_ is a practical way to enhance the electromechanical response of DEs. This can be achieved through three main strategies. A representative choice is using polarized rubber matrix such as polyacrylate rubber (AR) [14,15], with abundant ester groups providing strong dipolar polarization or grafting dipole functional groups onto the non-polar rubber molecular chains. The second strategy is to blend the DE matrix with conductive fillers such as carbon nanotubes [16], carbon nanospheres [17], and graphene [18]. Thanks to interfacial polarization and microcapacitor networks formed by sub-percolating conductive fillers, *ε*_r_ increases sharply as the filler content approaches the percolation threshold [19]. The drawback is that they also raise electrical conductivity, which markedly lowers *E*_b_ and increases dielectric loss (*ε*″), reducing energy conversion efficiency. The third strategy is to introduce high-*ε*_r_ ceramic fillers such as TiO_2_ [20,21] or BaTiO_3_ [22], a widely used and effective approach. However, their high surface energy promotes agglomeration, which degrades dispersion and easily forms defect sites, thereby harming electromechanical performance. Surface modification of the inorganic fillers can mitigate these issues by improving compatibility and dispersion [23]. For example, Yang et al. coated TiO_2_ with poly(dopamine) to strengthen interfacial adhesion with the rubber matrix, yielding simultaneous improvements in mechanical integrity and dielectric properties in polymer composites [24]. A disadvantage is that the cost of dopamine remains a barrier to large-scale industrial use [25].

In this study, we aim to investigate the potential application of DEAs in artificial bladders. For this purpose, a commercial chlorine cure-site AR was selected owing to its intrinsically high *ε*_r_ and good mechanical performance with its mechanical behavior close to that of porcine bladder tissue (Figure S1). This AR consists mainly of poly(ethyl acrylate) and contains approximately 5 wt% chlorine cure-site monomer [26]. These chlorine sites allow curing with trithiocyanuric acid (TCY) via nucleophilic substitution to form stable thioether crosslinking and a robust network [27]. However, the ester-rich, polar matrix requires tailored interfaces between the filler and the matrix to achieve good dispersion and electromechanical performance. In this work, TiO_2_ modified with a low-cost silane coupling agent (CA), γ-methacryloxypropyl trimethoxy silane, was employed to enhance the electromechanical performance of AR DEs (Figure 1b). Within AR composites, the purpose of using CA@TiO_2_ was to form a siloxane-rich interphase on the filler surface and create co-crosslinking bridges to the AR network via reactions of its double bond with thiol-bearing crosslinkers. This interfacial architecture improves compatibility and stabilizes dispersion. As a result, the interfacial area increases and interfacial polarization strengthens, yielding a higher *ε*_r_ at the same filler content. Meanwhile, fewer defects and a more uniform local field raise *E*_b_. At low content (10 wt%) of CA@TiO_2_, the *ε*_r_ increases, while maintaining low *Y* low dielectric loss and low hysteresis loss (high elasticity), the electromechanical sensitivity (*β* = *ε*_r_/*Y*) increases, delivering significantly enhanced actuated strain.

## 2. Experimental

### 2.1. Materials

Polyacrylate rubber (grade AR71) was supplied by Zeon Corporation (Tokyo, Japan). TiO_2_ nanoparticles with an average particle size of 15–25 nm were purchased from Beijing Dekedaojin Technology Co., Ltd. (Beijing, China). Trithiocyanuric acid (TCY, 99%, Shanghai Jinghai Chemical Co., Ltd., Shanghai, China), 2-mercaptobenzothiazole (BZ, 99%, Kemiou Chemical Co., Ltd., Tianjin, China), γ-methacryloxypropyl trimethoxy silane (CA, 99%, Aladdin, Shanghai, China) and anhydrous ethanol (≥99.7%, Beijing Chemical Works, Beijing, China) were used as received without further purification.

### 2.2. Preparation of CA@TiO_2_ Filler

TiO_2_ particles were first mixed with 5 wt% CA (dissolved in ethanol) using a high-speed mixer at a rotating speed of 30,000 r/min for 4 min, then placed in an oven and dried at 80 °C for 2 h. The pretreated TiO_2_ was subsequently washed three times with ethanol and dried again at 80 °C for 8 h to obtain CA@TiO_2_ filler.

### 2.3. Preparation of Composites

TiO_2_/AR or CA@TiO_2_/AR compounds containing TiO_2_ or CA@TiO_2_ (0, 10, 30, or 50 phr), TCY (0.25 phr, vulcanizing agent), BZ (0.5 phr, accelerator), and AR (100 phr) were prepared by physical mixing on a 6-inch two-roll mill at room temperature for 30 min, with detailed formulations provided in Table S1. The TiO_2_/AR and CA@TiO_2_/AR dielectric composite films were obtained by vulcanizing the corresponding compounds under a pressure of 15 MPa at 170 °C.

### 2.4. Characterization Methods

See the Supporting Information.

## 3. Results and Discussion

### 3.1. Structural Characterization of CA@TiO_2_

**Figure 2:**
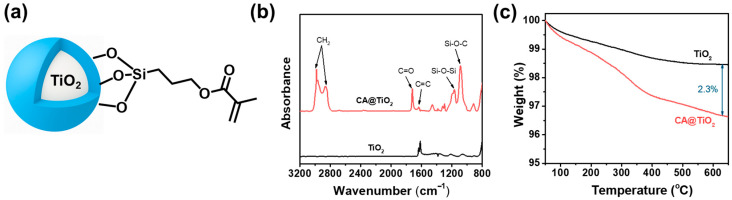
(**a**) Schematic representation of CA@TiO_2_ filler. (**b**) FTIR spectra and (**c**) TGA thermograms of pristine TiO_2_ and CA@TiO_2_ filler.

The CA-functionalized TiO_2_ filler is schematically illustrated in Figure 2a, while the corresponding FT-IR spectra of TiO_2_ and CA@TiO_2_ are presented in Figure 2b. Compared with the pristine TiO_2_ filler, the CA@TiO_2_ exhibits new characteristic peaks at 1162 cm^−1^ and 1197 cm^−1^, corresponding to the Si-O-Si bond stretching vibration of CA, as well as peaks at 2981 cm^−1^ and 2865 cm^−1^, which are attributed to -CH_2_- stretching vibrations from CA. In addition, new absorption peaks appear at 912 cm^−1^, 1085 cm^−1^, 1640 cm^−1^, and 1721 cm^−1^, representing the stretching vibrations of Si-OH, Si-O-C, C=C, and C=O groups of CA, respectively [28]. These results confirm that CA was successfully grafted onto the TiO_2_ surface. TGA was used to determine the grafting amount of CA on the TiO_2_ surface. As shown in Figure 2c, when heated from 200 to 650 °C, pristine TiO_2_ and CA@TiO_2_ show weight losses of 1.5% and 3.8%, respectively. Based on Equation (S1), the grafting density on TiO_2_ was calculated as 0.12 mmol/g.

### 3.2. Microstructure of AR Composites

**Figure 3:**
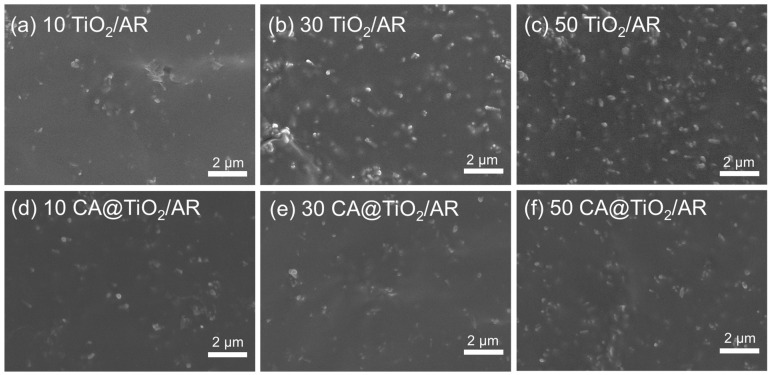
SEM micrographs of AR composites filled with (**a**) 10 phr TiO_2_, (**b**) 30 phr TiO_2_, (**c**) 50 phr TiO_2_, (**d**) 10 phr CA@TiO_2_ (**e**) 30 phr CA@TiO_2_ and (**f**) 50 phr CA@TiO_2_.

The morphology of TiO_2_/AR and CA@TiO_2_/AR composites was examined by SEM, and the results are shown in Figure 3. The unmodified TiO_2_ exhibits poor dispersion, with pronounced particle agglomeration due to the large surface energy mismatch between TiO_2_ and the AR matrix. In contrast, CA@TiO_2_ fillers are dispersed more uniformly in the AR matrix. This improvement arises from CA serving as a co-crosslinking bridge through interfacial covalent bonding, which enhances the compatibility between TiO_2_ particles and the AR matrix.

### 3.3. Mechanical Properties of AR Composites

**Figure 4:**
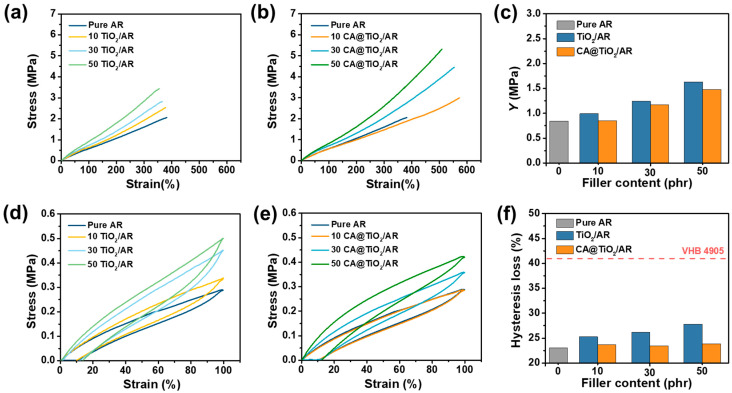
Stress–strain curves of (**a**) TiO_2_/AR and (**b**) CA@TiO_2_/AR composites. (**c**) *Y* of all AR composites. Cyclic stress–strain curves of (**d**) TiO_2_/AR and (**e**) CA@TiO_2_/AR composites. (**f**) Hysteresis loss of all AR composites.

The stress–strain curves of TiO_2_/AR and CA@TiO_2_/AR composites are shown in Figure 4a and Figure 4b, respectively, and the mechanical properties of all AR composites are summarized in Table S2. The tensile strength of the AR composites exceeds that of pure AR and increases with filler content, owing to the reinforcing effect of rigid fillers. Specifically, the filler particles can promote stress transfer from the flexible AR matrix to the rigid inorganic filler during stretching. At the same filler loading, the CA@TiO_2_/AR composites exhibit higher tensile strength and elongation at break than the TiO_2_/AR composites, owing to the better filler dispersion and stronger filler–matrix interaction, which reduce stress concentration and improve load transfer efficiency.

The *Y* of the AR composites is shown in Figure 4c. At 10 phr TiO_2_ or CA@TiO_2_, *Y* differs only slightly from pure AR, indicating that the filler network structure has not been formed yet under low filler fraction. At higher contents, *Y* increases significantly because of the formation of filler networks that stiffen the matrix. In addition, at the same filler content, the *Y* of CA@TiO_2_/AR composites is lower than that of TiO_2_/AR composites, which is attributed to better dispersion and a more compliant siloxane interphase that reduces the compactness of the network structure.

In addition, low hysteresis loss (also known as viscoelastic hysteresis) is an important evaluation criterion for elasticity of DEs. The cyclic stress–strain curves of TiO_2_/AR and CA@TiO_2_/AR composites are shown in Figure 4d and Figure 4e, respectively, and the hysteresis loss of all AR composites are summarized in Figure 4f. Whether or not the fillers are surface-modified, the hysteresis loss of AR composites increases with TiO_2_ content, which is attributed to increased interfacial friction, interfacial sliding, and the strengthening of filler network at higher loadings. At the same filler content, CA@TiO_2_/AR composites show a lower hysteresis loss than TiO_2_/AR composites due to a robust interphase and the better dispersion of TiO_2_. These interfacial interactions provide stronger anchoring points and suppress interfacial slippage, facilitating chain recovery and reducing energy dissipation. The hysteresis loss of the 10 CA@TiO_2_/AR composite is comparable to that of pure AR (23%), indicating the high elasticity being maintained. Moreover, the hysteresis loss of all AR composites remains below 28%, well below that of VHB 4905 (41%), a widely used acrylic-based DE [29].

### 3.4. Dielectric Properties of AR Composites

**Figure 5:**
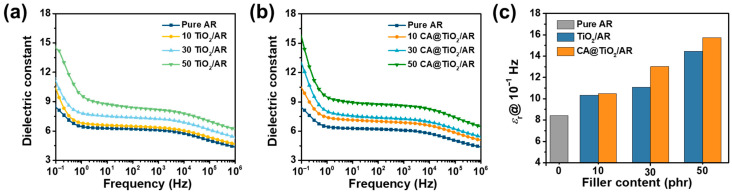
*ε*_r_ versus frequency of (**a**) TiO_2_/AR and (**b**) CA@TiO_2_/AR composites. (**c**) *ε*_r_ @ 10^−1^ Hz of all AR composites.

The *ε*_r_ of TiO_2_/AR and CA@TiO_2_/AR composites over 10^−1^–10^6^ Hz is shown in Figure 5a and Figure 5b, respectively. The *ε*_r_ of all composites decreases with increasing frequency, indicating strong frequency dependence arising from dipolar relaxation. At low frequencies, dipoles associated with the ester side groups in AR can follow the alternating field, yielding high polarization and therefore higher *ε*_r_. As the frequency increases, dipole reorientation cannot keep pace, polarization diminishes, and *ε*_r_ falls. In addition, *ε*_r_ increases with TiO_2_ content, whether or not the fillers are surface-modified, because of interfacial polarization between the dielectric filler and the rubber matrix.

More importantly, the *ε*_r_ @ 10^−1^ Hz of CA@TiO_2_/AR composites is higher than that of TiO_2_/AR composites at the same filler content (Figure 5c). This enhancement mainly originates from the improved dispersion of CA@TiO_2_ in the AR matrix, which increases the number of effective polymer-filler phase interfaces and thereby enhances the overall contribution of interfacial polarization. Notably, this trend is different from our previous reported SiO_2_/SiR system, where the *ε*_r_ @ 10^−1^ Hz of CA@SiO_2_/SiR composites is lower than that of SiO_2_/SiR composites at the same filler loading [30]. Although surface modification in the CA@SiO_2_/SiR system improves dispersion and enlarges the total interfacial area, the CA layer weakens the interfacial polarization at each individual SiO_2_-SiR interface, leading to an overall decrease in *ε*_r_. These opposite trends indicate a trade-off between filler dispersion and interfacial structure in determining the overall interfacial polarization contribution. As a result, the 50 CA@TiO_2_/AR composite shows the highest *ε*_r_ @ 10^−1^ Hz of 15.73, which is 1.87 times than that of neat AR (8.43).

**Figure 6:**
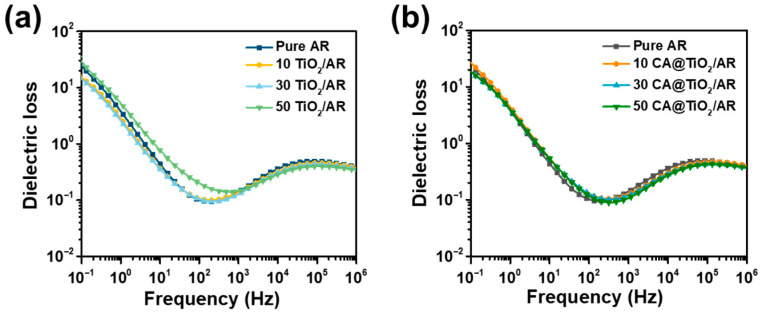
*ε*″ versus frequency at room temperature of (**a**) TiO_2_/AR and (**b**) CA@TiO_2_/AR composites.

Consistent with the frequency dependence of *ε*_r_, the *ε*″ of all composites also exhibits an obvious frequency-dependent behavior (Figure 6). At low frequencies, the *ε*″ is relatively high, mainly because interfacial polarization and dipolar relaxation can fully respond to the slowly varying electric field, resulting in greater energy dissipation. With increasing frequency, the *ε*″ gradually decreases because the dipoles and interfacial charges can no longer follow the rapid alternation of the electric field in a timely manner. Therefore, the polarization process becomes progressively suppressed, leading to reduced *ε*″ at higher frequencies.

**Figure 7:**
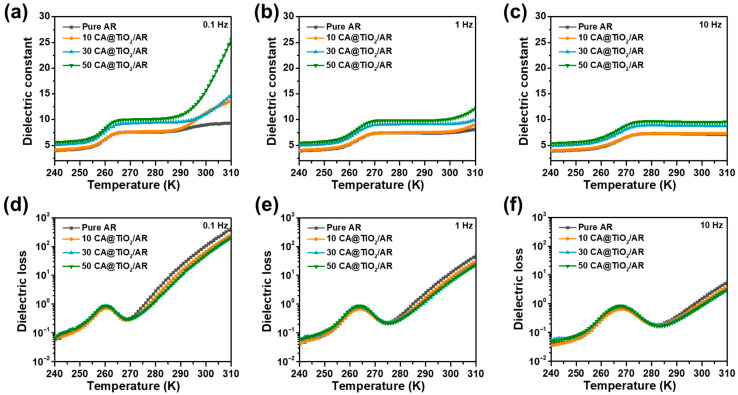
*ε*_r_ versus temperature of CA@TiO_2_/AR composites at (**a**) 0.1 Hz, (**b**) 1 Hz, and (**c**) 10 Hz; *ε*″ versus temperature of CA@TiO_2_/AR composites at (**d**) 0.1 Hz, (**e**) 1 Hz, and (**f**) 10 Hz.

To clarify the physical origin of the dielectric properties, the dielectric relaxation behavior of pure AR and CA@TiO_2_/AR composites was investigated over a wide temperature range at three low frequencies (0.1, 1, and 10 Hz), which are relevant to the expected operating conditions of the artificial bladder (Figure 7) [31,32]. As shown in Figure 7a–c, the *ε*_r_ of all samples gradually increases with increasing temperature. In addition, the composites with higher CA@TiO_2_ content exhibit higher *ε*_r_, especially at low frequencies. This behavior can be attributed to the enhanced interfacial polarization and the increased mobility of polar groups at elevated temperature. Meanwhile, as shown in Figure 7d–f, all samples exhibit a broad temperature-dependent dielectric-loss relaxation feature. However, no obvious change in peak position or peak intensity with increasing filler content, suggesting that the introduction of CA@TiO_2_ does not significantly alter the dipolar relaxation behavior of the AR matrix.

**Figure 8:**
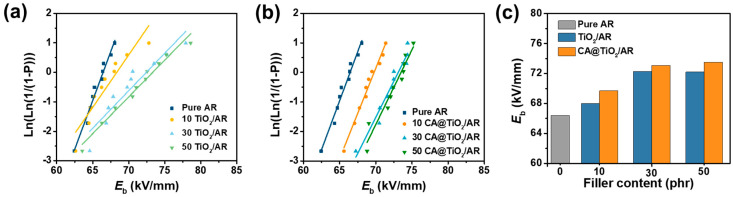
*E*_b_ in electric insulating oil calculated from *Weibull* distribution of (**a**) TiO_2_/AR and (**b**) CA@TiO_2_/AR composites, and (**c**) Comparison of *E*_b_ among all AR composites.

In addition, the *E*_b_ is a key parameter that limits the maximum actuated strain, as indicated by Equation (1). As shown in Figure 8a,b, *E*_b_ of all composites increases with TiO_2_ content for both pristine and CA-modified fillers, consistent with previous reports [22,33]. This can be attributed to the introduction of rigid TiO_2_ particles, which restrict the segmental mobility of AR chains and increase the tortuosity of charge transport pathways, thereby suppressing space charge migration and electrical treeing and improving the local electric field distribution [34]. Moreover, CA@TiO_2_/AR composites show a higher electrical breakdown field than TiO_2_/AR composites at the same filler content, which is attributed to improved filler dispersion and fewer defect sites, and the formation of an insulating layer on the filler surface (Figure 8c). Accordingly, the 50 CA@TiO_2_/AR composite shows the highest *E*_b_ of 73.5 kV/mm, which is 1.11 times that of neat AR (66.4 kV/mm).

### 3.5. Actuation Performance of Composite

**Figure 9:**
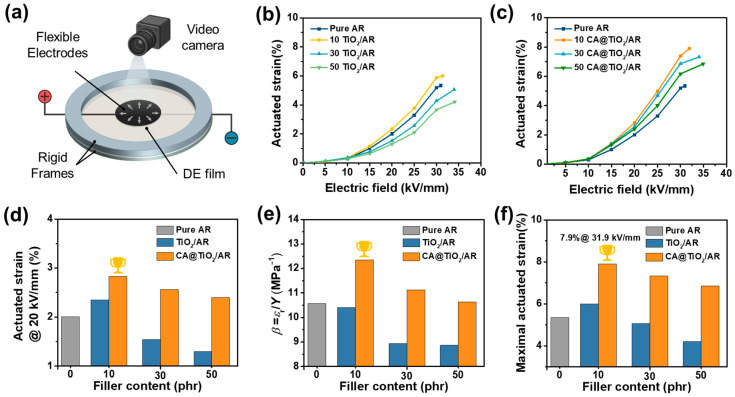
(**a**) Schematic of actuated strain measurement using a circular in-plane actuator. Actuated strain versus electrical field of (**b**) TiO_2_/AR composites and (**c**) CA@TiO_2_/AR composites. (**d**) Actuated strain @ 20 kV/mm, (**e**) electromechanical sensitivity, and (**f**) maximum actuated strain of all AR composites.

Actuated strain is central to evaluating actuation performance. We measured it with a circular in-plane actuator without any pre-stretch (Figure 9a). The actuated strain at different electric fields for TiO_2_/AR and CA@TiO_2_/AR composites is shown in Figure 9b and Figure 9c, respectively, and the corresponding actuation performance is summarized in Table S3. For all samples, the actuated strain increases with electric field and follows a quadratic dependence on the electric field, as shown in Equation (1). At a given electric field (e.g., 20 kV/mm), CA@TiO_2_/AR composites exhibit larger actuated strain than TiO_2_/AR composites at the same filler content, with the 10 CA@TiO_2_ composite showing the largest actuated strain (Figure 9d). These results arise from the differences in electromechanical sensitivity (*β* = *ε*_r_/*Y*), consistent with Equation (1). As shown in Figure 9e, CA@TiO_2_/AR composites have higher *β* than TiO_2_/AR composites at the same filler content, because they combine a higher *ε*_r_ with a lower *Y*. Meanwhile, *β* in CA@TiO_2_/AR composites first increases and then decreases with filler content. At the 10 phr CA@TiO_2_ loading, *ε*_r_ increases with only a slight change in *Y*, leading to the highest *β*. By comparison, at higher filler contents, *ε*_r_ still increases while Y rises more rapidly, leading to a drop in *β*. As a result, owing to its highest *β* and a relatively high *E*_b_, the 10 CA@TiO_2_/AR composite reaches the maximal actuation strain of 7.9% at 31.9 kV/mm, which is 1.48 times that of pure AR and 1.32 times that of the 10 TiO_2_/AR composite (Figure 9f).

## 4. Conclusions

In summary, we demonstrated a simple and effective route to enhance the electromechanical performance of AR DEs by incorporating silane-functionalized TiO_2_. Compared with pristine TiO_2_, CA@TiO_2_ forms a siloxane-rich interphase and covalent bridges to the AR network, which improves compatibility, stabilizes dispersion, and thus increases the number of effective polymer-filler phase interfaces and thereby enhances the overall contribution of interfacial polarization. Therefore, at the same filler content, CA@TiO_2_/AR composites show lower *Y*, higher tensile strength, lower hysteresis loss (indicating good elasticity), higher *ε*_r_, and higher *E*_b_. Overall, the electromechanical sensitivity (*β* = *ε*_r_/*Y*) of AR composites reaches a maximal value at 10 phr CA@TiO_2_ and declines at higher loadings as *Y* increases more rapidly than *ε*_r_. As a result, the 10 CA@TiO_2_/AR composite exhibits a maximal actuated strain of 7.9% at 31.9 kV/mm without pre-stretch, which is 1.48 times that of pure AR and 1.32 times that of the 10 TiO_2_/AR composite. This low-cost surface modification strategy is broadly applicable for improving the electromechanical performance of DE composites and provides a practical route to high-performance, scalable soft actuators with potential use in artificial bladder applications.

## Data Availability

The original contributions presented in this study are included in the article/Supplementary Material. Further inquiries can be directed to the corresponding authors.

## Supplementary Materials

The following supporting information can be downloaded at: https://www.mdpi.com/article/10.3390/polym18070872/s1, Figure S1: Stress-strain curves of porcine bladder in the longitudinal and transverse directions; Table S1: Experimental formulation of AR composites; Table S2: Mechanical and dielectric properties of AR composites; Table S3: Electrical actuation performance of AR composites.
