# Supplementary material for: Enhanced Electromechanical Performance of Dielectric Elastomer by Co-Crosslinking of Silane-Functionalized TiO2 with Polyacrylate
Source: Polymers (Basel). 2026 Apr 1;18(7):872. doi: 10.3390/polym18070872 (PMC13075199; doi:10.3390/polym18070872)
Supplement: Supplementary file 1 [file polymers-18-00872-s001.zip › polymers-4154083-supplementary.pdf]

Enhanced Electromechanical Performance of Dielectric Elastomer by Co-Crosslinking of Silane-Functionalized TiO_2 with Polyacrylate

Lingxiao Peng[1,†], Wenjie Si[1,†], Yuhui He[2], Nanying Ning[1,*] and Jianfeng Wang[2,*]

1. State Key Laboratory of Organic-Inorganic Composites, Beijing University of Chemical Technology, Beijing, 100029, China.

2. Department of Urology, China-Japan Friendship Hospital, Beijing, 100029, China.

† These authors contributed equally, and they should be regarded as co-first authors.

* Corresponding authors.

E-mail addresses: ningny@mail.buct.edu.cn (Nanying Ning);

zryhyy1@126.com (Jianfeng Wang).

Part I: Characterization methods

Fourier transform infrared spectroscopy (FT-IR): The structural analysis of pristine TiO_2 and CA@TiO_2 were characterized by Fourier transform infrared spectroscopy (Tensor 27, Bruker Optik, Germany) from 3200 to 800 cm^{-1} in transmission mode with a disc of KBr.

Thermogravimetric analysis (TGA): The thermostability of pristine TiO_2 and CA@TiO_2 was evaluated by thermogravimetric analyzer (TGA55, TA Instruments, USA). Samples were heated from 40 °C to 650 °C at a heating rate of 10 °C/min under nitrogen flow rate of 25 mL/min. The grafting density of coupling agent on TiO_2 particles was calculated by:

$$\text{Grafting density (mmol/g)} = \frac{W_{250-650}\times 10^{3}}{(100-W_{250-650})\times M} \quad \text{(S1)}$$

where M is the molecular weight of coupling agent (γ-methacryloxypropyl trimethoxy silane, 248.4 g/mol) and $W_{250-650}$ is the weight loss between 250 and 650 °C.

Scanning electron microscope (SEM): AR composites were thoroughly cryo-treated in liquid nitrogen and immediately fractured to expose the cross-section. The fracture surfaces were mounted face-up on SEM stubs using conductive tape and sputter-coated with gold to ensure surface conductivity. The microstructures of AR composites were examined using a scanning electron microscope (SEM, Hitachi S-4800) operated at an accelerating voltage of 5.0 kV to assess the dispersion of fillers within the rubber matrix.

Mechanical properties: The tensile tests and cyclic stress-strain tests of the AR composites were performed on dumbbell-shaped samples (length × width × thickness:20 × 4× 1 mm^3) using a tensile apparatus (Instron 5567, USA) at room temperature with a tensile rate of 500 mm/min. The elastic modulus of the samples was determined by the slope of the stress-strain curve at 10% strain. For the cyclic stress-strain test, the sample was stretched to a constant strain of 200% and then carried out loading process without relax time at same speed. The hysteresis loss of the AR composites was determined from the cyclic stress-strain curves.

Dielectric properties: The dielectric properties of AR composites were measured by a broadband dielectric spectrometer (BDS, Concept 40, Novocontrol GmbH, Germany) over the frequency range of 10^{-1} Hz to 10^{6} Hz at room temperature. Temperature-dependent dielectric measurements were carried out from 240 K to 310 K with a Quatro temperature control system (Novocontrol GmbH, Germany) under nitrogen atmosphere. The samples have a diameter of 20 mm and a thickness of 0.5 mm.

Electric breakdown strength (E_b): The AR composites with the thickness (*d*) of approximately 0.3 mm, sandwiched between two copper electrode with 20 mm diameter, were immersed in electric insulating oil before being tested. The voltage was supplied by a high-voltage direct current generator (DTZH-60, Wuhan Dotek Electric) under a ramp rate of 1000 V/s until electric breakdown voltage (U_b). The dielectric breakdown strength (E_b) was calculated by:

$$E_{\mathrm{b}} = \frac{U_{\mathrm{b}}}{d} \quad \text{(S2)}$$

Each sample was tested using ten specimens, and the resulting values were reported after conducting Weibull analysis.

Electrical Actuation Performance: The actuated strain of AR composites were measured using a circular in-plane actuator without any pre-stretch. AR composites with a thickness of approximately 0.3 mm were prepared and fixed in a rigid circular frame (inner diameter 30 mm, outer diameter 50 mm). Circular carbon grease electrodes with a diameter of 15 mm were coated on both surfaces of the DE film using a mask method. The electrode regions on both sides were connected to the positive and negative terminals of a high-voltage DC generator (DTZH-60, Wuhan Dotek Electric Co., Ltd., China) through copper foil tape. The applied voltage was increased from 0 kV at a rate of 100 V/s until dielectric breakdown occurred. During the test, a video camera (Canon IXUS 80, Japan) was used to record the variation in electrode area under different voltages. The photographs were processed using

Photoshop software, where the number of pixels in the electrode region (fixed focal length and resolution) was used to calculate the area. The planar actuated strain S_a was determined according to:

$$S_a = \frac{A - A_0}{A_0} \times 100\% \quad \text{(S3)}$$

where A is the electrode area under an applied voltage and A_0 is the initial electrode area at 0 kV.

Part II: Figure and Tables

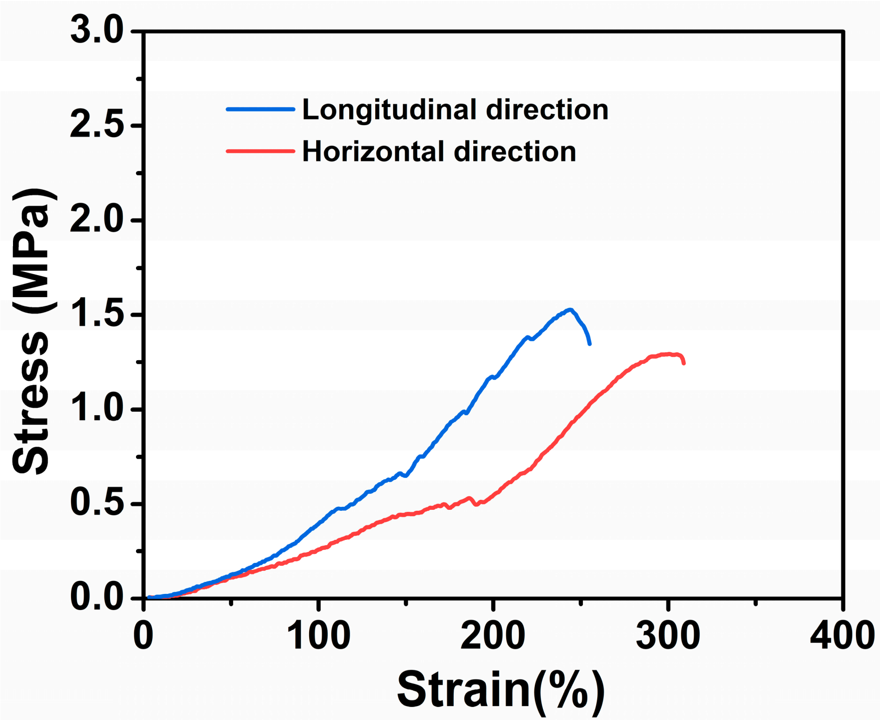

Figure S1. Stress-strain curves of porcine bladder in the longitudinal and transverse directions.

Table S1. Experimental formulation of AR composites.

Samples	AR71	TCY	BZ	TiO_2	CA
Pure AR	100	0.25	0.5	0	0
10 TiO_2/AR	100	0.25	0.5	10	0
30 TiO_2/AR	100	0.25	0.5	30	0
50 TiO_2/AR	100	0.25	0.5	50	0
10 CA@TiO_2/AR	100	0.25	0.5	10	0.5
30 CA@TiO_2/AR	100	0.25	0.5	30	1.5
50 CA@TiO_2/AR	100	0.25	0.5	50	2.5

Table S2. Mechanical and dielectric properties of AR composites.

Samples	Modulus (MPa)	Elongation at Break (%)	Tensile strength (MPa)	Dielectric constant @10^{-1}Hz
Pure AR	0.84	382	2.06	8.43
10 TiO_2/AR	0.99	378	2.53	10.33
30 TiO_2/AR	1.24	366	2.82	11.09
50 TiO_2/AR	1.63	355	3.44	14.46
10 CA@TiO_2/AR	0.85	572	2.99	10.49
30 CA@TiO_2/AR	1.17	553	4.45	13.02
50 CA@TiO_2/AR	1.48	509	5.31	15.73

Table S3. Electrical actuation performance of AR composites.

Samples	electromechanical sensitivity (MPa^{-1})[a]	Nominal breakdown strength (kV/mm)[b]	Maximal actuated strain (%)
Pure AR	10.00	31.0	5.34
10 TiO_2/AR	10.40	31.3	6.00
30 TiO_2/AR	8.94	33.9	5.06
50 TiO_2/AR	8.87	34.1	4.21
10 CA@TiO_2/AR	12.34	31.9	7.90
30 CA@TiO_2/AR	11.13	34.0	7.32
50 CA@TiO_2/AR	10.63	34.9	6.85

[a] Electromechanical sensitivity (β) is calculated as ε_r/Y, where ε_r is obtained from the ε_r at 10^{-1} Hz and Y is the elastic modulus of the samples. The corresponding parameters are shown in Table S2.

[b] Nominal breakdown strength was measured in air at room temperature (≈25 ^{o}C) with relative humidity below 30%.
